# Lifestyle factors or protective behaviors: which are more strongly associated with upper respiratory tract infections after COVID-19? A cross-sectional study of 1,535 Chinese college students

**DOI:** 10.3389/fpubh.2026.1958833

**Published:** 2026-09-23

**Authors:** Chenyun He, Ting Li, Yanhua Li, Jie Ning, Chunyan Xia

**Affiliations:** 1 Health Center, Changzhou University, Changzhou, China; 2 Changzhou No. 7 People’s Hospital, Changzhou, China

**Keywords:** cross-sectional study, life style, risk reduction behavior, SARS-CoV-2, students, upper respiratory tract infection (URTI)

## Abstract

**Background:**

Respiratory pathogens have resurged following the COVID-19 pandemic. College students are socially active and often live or study in congregate settings, which may place them at continued risk of upper respiratory tract infection (URTI).

**Objective:**

To compare the strength of the associations of lifestyle factors and protective behaviors with the number of URTI episodes among college students in the post-COVID-19 period.

**Methods:**

We conducted a cross-sectional survey from June 1 to June 30, 2024, among 2,100 students at six universities in southeastern China. Students were eligible if their first SARS-CoV-2 infection had occurred at least 1 year before the survey. We collected the number of URTI episodes from June 1, 2023, to June 1, 2024, together with demographic, lifestyle, and protective-behavior data. The analysis included 1,535 valid questionnaires. Hierarchical negative binomial regression was used. Demographic variables were entered in the baseline model, after which lifestyle factors and protective behaviors were added sequentially. Their entry order was then reversed to compare the relative contribution of each variable group.

**Results:**

When entered first into the baseline model, lifestyle factors produced a greater improvement in model fit than did protective behaviors (Δ*χ*^2^/df, 7.34 vs. 2.48; AIC, 4981.4 vs. 5067.9). Female sex, higher BMI categories, chronic diseases, COVID-19 vaccination ≥2 doses, daytime sleepiness (IRR, 1.768–2.324), sedentary, vigorous-intensity physical activity were associated with more URTI episodes, whereas sleep duration of at least 7 h and moderate-intensity physical activity were associated with fewer episodes (all *p* < 0.05). Among protective behaviors, only natural ventilation showed a consistent inverse association (IRR: 0.425–0.475; *p* < 0.05). Most other behaviors, including handwashing before meals, maintaining 1 m distance, avoiding touching the eyes/mouth/nose and public surfaces, and disinfecting phones and keys were not significantly associated with URTI episodes.

**Conclusion:**

Lifestyle factors were more strongly associated with URTI episodes than protective behaviors among college students in the post-COVID-19 period. These findings suggest that modifiable lifestyle factors, including sleep, physical activity, and sedentary behavior, as well as natural ventilation, may be relevant targets for campus health strategies.

## 1 Introduction

SARS-CoV-2 can reduce lymphocyte counts and impair their function (1–3). In some people who have recovered from COVID-19, immune alterations may persist (2). These immunological changes may increase susceptibility to other respiratory pathogens. Respiratory infections have resurged in China and other countries following the pandemic (4–7). China has also experienced an alternating seasonal pattern of summer COVID-19 waves and winter influenza epidemics. College students are socially active and often live in congregate settings (8); therefore, they remain at continued risk of upper respiratory tract infection (URTI).

At the end of 2022, China experienced a massive wave of SARS-CoV-2 infections. In early 2023, the mask mandate in public places was canceled, and most social containment measures were lifted. Protective behaviors among college students then diverged rapidly. By June 2024, some students still adhered to mask wearing indoors and practicing hand hygiene, while others had completely dropped these practices. This behavioral divergence provided a unique window to examine the relative associations of lifestyle factors and protective behaviors with URTI episodes.

Previous studies have shown that lifestyle factors, such as sleep and physical activity, and protective behaviors, such as mask wearing and handwashing, may influence the occurrence of respiratory infections (9–12). However, most studies have examined only one group of factors or have included some variables from both groups as covariates. Direct comparisons of lifestyle factors and protective behaviors as complete variable groups remain limited. It is therefore unclear which group has the stronger overall association with URTI episodes. In addition, the entry order of variable groups may affect model fit indices. This methodological issue has rarely been systematically examined when comparing different groups of factors.

We constructed hierarchical negative binomial regression models. Lifestyle factors and protective behaviors were entered sequentially, and we repeated the analysis after reversing their entry order. We compared changes in the Akaike information criterion (AIC), the chi-square statistic per degree of freedom (*χ*^2^/df), and incidence rate ratios (IRRs). This allowed us to assess the relative associations of the two variable groups with URTI episodes and to identify important factors within each group. We hypothesized that lifestyle factors would show stronger associations with URTI episodes than protective behaviors. The findings may inform targeted public health strategies on university campuses.

## 2 Methods

### 2.1 Study participants

This study was conducted from June 1 to June 30, 2024, in southeastern China, a region with a high concentration of universities. Eligible participants were college students in China. The inclusion criteria were: (1) a history of SARS-CoV-2 infection, defined by either (a) a positive antigen or nucleic acid amplification test, or (b) compatible clinical symptoms (e.g., fever, cough, sore throat, or loss of taste or smell) during peak periods of community transmission (November 2022–January 2023), together with a positive test result in a close contact (e.g., a roommate, family member, or classmate); (2) first infection on or before June 1, 2023. The exclusion criteria were: (1) age >30 years, to minimize heterogeneity in physiological status; and (2) current enrollment in a Master’s or Doctoral program, as their studying, living, and social environments differ substantially from those of undergraduate students.

#### 2.1.1 Sample size estimation

Negative binomial regression was used to identify factors associated with the number of URTI episodes. Because no universally accepted sample-size formula is available for this model, we applied an events-per-variable (EPV) approach. We used a conservative threshold of 15 events per variable, which is higher than the conventional threshold of 10 (13, 14). The planned model included 21 independent variables and therefore required at least 315 positive events. A positive event was defined as at least one URTI episode during the previous year.

Pilot data from 100 students were used to estimate the required sample. In the pilot study, 83.0% of participants met the eligibility criterion of a first SARS-CoV-2 infection at least 1 year earlier. Among eligible participants, 85.5% provided valid responses, and 45.0% of valid respondents reported at least one URTI episode during the previous year. The required effective sample was therefore 700 participants (315/0.45). After adjustment for the valid-response rate and eligibility rate, 987 questionnaires were required (700/0.855/0.830). We then applied a design effect of 1.8, a conservative value for a multicenter cross-sectional survey (15, 16), resulting in a final target of approximately 1,692 questionnaires.

We distributed 2,100 questionnaires based on the sampling plan of six universities with 350 students per institution. This number exceeded the estimated minimum sample size and was expected to provide sufficient statistical precision.

#### 2.1.2 Ethics approval and informed consent

This study was approved by the Ethics Committee of Changzhou University (Approval No. 2024102202). Prior to survey administration, all students present in the classroom were provided with an informed consent statement on the first page of the electronic questionnaire. The statement detailed the study objectives, the voluntary nature of participation, and the confidentiality of their responses. Participants who consented selected “agree” to proceed to the formal questionnaire, while those who did not consent selected “disagree” and were automatically redirected to the survey termination page.

### 2.2 Methods

#### 2.2.1 Questionnaire and variable definitions

Data were collected using an investigator-developed questionnaire titled “Lifestyle and Protective Behaviors Associated With URTI Episodes in College Students.” The questionnaire covered demographic characteristics, the history and timing of SARS-CoV-2 infection, URTI episodes, lifestyle factors, and protective behaviors.

##### 2.2.1.1 SARS-CoV-2 infection, COVID-19 vaccination doses and URTI episodes

SARS-CoV-2 infection status was self-reported according to the inclusion criteria defined above. Participants indicated whether they had ever been infected and, if so, reported the month and year of their first infection. COVID-19 vaccination doses received before September 15, 2022, were categorized as ≤1 and ≥2.

A URTI episode was defined as the acute onset of at least two of the following symptoms: sore throat, nasal congestion, rhinorrhea, or cough (17). The definition encompassed both self-reported and clinically diagnosed episodes, with the latter verified against medical records whenever accessible. The definition applied regardless of whether the suspected pathogen was SARS-CoV-2 or another respiratory virus. Symptoms were required to persist for more than 24 h, and separate episodes were required to be divided by at least 48 symptom-free hours. An episode that resolved within 24 h after medication was still counted. An illness that began with URTI symptoms and later progressed to physician-diagnosed bronchitis or pneumonia was counted as one episode. Episodes clearly attributable to allergic rhinitis or asthma were excluded. To assist differentiation, participants were also asked whether at least one reported episode was accompanied by fever (≥37.3 °C) or myalgia.

To reduce recall bias, URTI episodes during the previous 12 months were assessed in two stages. Participants first provided an overall estimate, which served only as a recall prompt and was not used in the analysis. They then reported episodes separately for each 3-month period. The annual total was calculated as the sum of the four quarterly counts.

##### 2.2.1.2 Demographic characteristics

The demographic variables were age (17–18 vs. 19–30 years), sex, height, weight, and chronic diseases. Height and weight were used to calculate body mass index (BMI), which was classified according to Chinese criteria as underweight, normal weight, overweight, or obesity. Chronic diseases included hypertension, type 2 diabetes, asthma, chronic obstructive pulmonary disease, kidney disease, hematologic malignancy, and HIV/AIDS. A chronic disease was recorded when the participant reported a physician diagnosis.

##### 2.2.1.3 Lifestyle factors

Lifestyle factors were defined as habitual, long-term behavioral patterns that are relatively stable over time and are not directly contingent on the perceived threat of infection.

- *Smoking*: Participants were categorized as current smokers or nonsmokers; former smokers were classified as nonsmokers (18).
- *Alcohol use*: Participants were categorized as current drinkers or nondrinkers; former drinkers were classified as nondrinkers (19).
- *Sleep*: Sleep measures included total sleep duration, including naps (hours/day), and the frequency of daytime sleepiness (0 days/week, <1 day/week, 1–2 days/week, or ≥3 days/week).
- *Sedentary behavior*: Sedentary behavior was defined as waking behavior performed while sitting or reclining with an energy expenditure of ≤1.5 metabolic equivalents and a total duration of ≥8 h/day (20).
- *Physical activity*: According to the Chinese Sport Science Society standard (T/CSSS 002—2023), physical activity intensity was classified into three levels: light (1.6–2.9 METs), moderate (3.0–5.9 METs), and vigorous (≥6.0 METs). Representative examples of light-intensity activities were walking and planks; moderate-intensity activities included yoga, brisk walking, cycling on flat ground, recreational badminton, recreational basketball, and table tennis; and vigorous-intensity activities included jogging, stair climbing, uphill cycling, rope skipping, competitive badminton, and competitive basketball.

##### 2.2.1.4 Protective behaviors during the previous 12 months

Protective behaviors were defined as situation-specific, modifiable actions adopted in response to the perceived risk of infection, particularly during the pandemic. Response scales differed by item. Most behaviors were assessed on a five-point scale ranging from almost never to always. Disinfecting phones and keys was measured as weekly frequency (0 to ≥7 times/week). Natural ventilation was measured as daily frequency (almost never to ≥2 times/day), with each ventilation session lasting at least 30 min.

Measures intended to reduce contact transmission included handwashing before meals and after restroom use, avoiding touching the eyes, mouth, or nose, avoiding contact with public surfaces, and disinfecting phones and keys.

Measures intended to reduce respiratory transmission included mask wearing indoors, such as classrooms with closed windows and elevators; maintaining 1-meter distance when feasible; and opening windows for natural ventilation.

#### 2.2.2 Survey procedure

A stratified random sampling strategy was employed to account for the heterogeneity of university types in Jiangsu Province. Based on Ministry of Education classification, universities were divided into three strata: comprehensive/polytechnic, liberal arts, and vocational colleges. From each stratum, two institutions were randomly selected using a random number table, yielding a total of six universities. Within each university, 350 students were randomly sampled, yielding 2,100 distributed questionnaires. Through questionnaire-based screening, participants who reported a first SARS-CoV-2 infection at least 1 year before the survey were enrolled in the final analytical sample. The survey was conducted using the “Wen Juan Xing” platform in classroom settings from June 1 to June 30, 2024. Trained investigators read standardized instructions emphasizing anonymity and the absence of “right or wrong” answers to reduce social desirability bias. Students who selected “agree” then completed the questionnaire independently and quietly, with on-site submission.

#### 2.2.3 Quality control

Quality-control procedures were applied throughout the study. During questionnaire design, validation items were included to identify random responses. During administration, completion time was recorded. Pilot data indicated that careful completion required approximately 90 s; questionnaires completed in less than 90 s and with failed validation items were flagged. During data cleaning, two researchers independently reviewed the responses and excluded questionnaires with patterned answers, such as the same response for all scale items. We excluded 317 questionnaires from students who had never been infected or whose first infection occurred less than 1 year before the survey, leaving 1,783 eligible questionnaires. After quality control, 1,535 valid questionnaires remained, corresponding to an effective response rate of 86.1% (1,535/1,783). The reliability and validity of the protective behavior scale were also assessed. The 8-item scale showed good internal consistency, with a Cronbach’s α of 0.853. Exploratory factor analysis supported its construct validity, identifying two factors (KMO = 0.879; Bartlett’s *p* < 0.001) that explained 62.60% of the variance, with all items loading above 0.4 on their respective factors.

### 2.3 Statistical analysis

Data cleaning, statistical analysis, and figure preparation were performed using SPSS 25.0 and R 4.2.2. Categorical variables are reported as counts and percentages. Because the number of URTI episodes was not normally distributed (Shapiro–Wilk test, *p* < 0.05), it is summarized as the median and interquartile range. Between-group comparisons were conducted using the Mann–Whitney U test for two-group comparisons and the Jonckheere–Terpstra test for ordered trends.

Hierarchical negative binomial regression was used to examine the associations of lifestyle factors and protective behaviors with URTI episodes (21, 22). Model 1 included demographic variables (sex, age, BMI, chronic diseases and COVID-19 vaccination doses). Model 2 additionally included lifestyle factors (sleep duration, daytime sleepiness, physical activity, sedentary behavior, smoking, and drinking). Model 3 further included protective behaviors (hand hygiene, mask wearing indoors, physical distancing, avoidance of contact with the eyes/mouth/nose and public surfaces, disinfecting phones and keys, and ventilation). The likelihood ratio test (LRT) was used to compare the goodness-of-fit between adjacent nested models. This allowed us to evaluate the changes in model fit with the sequential inclusion of variable groups.

The number of URTI episodes was a count outcome, with a mean of 1.52 and variance of 4.07 (variance-to-mean ratio = 2.68), indicating overdispersion. We therefore used negative binomial regression as the primary analytic approach, implemented with the MASS package in R. This model relaxes the Poisson assumption of equality between the mean and variance by allowing for unobserved heterogeneity in individual susceptibility. In Model 3, the estimated dispersion parameter (*θ*) was 0.786 (standard error, 0.061), confirming the presence of overdispersion and supporting the use of the negative binomial model. To ensure robust inference in the presence of minor model misspecification, we used heteroskedasticity-robust standard errors (HC3) to estimate 95% CIs and *p-*values via the sandwich package in R (23). Because 53.0% of participants reported no URTI episodes, we also fitted hurdle models (24). The results were generally consistent with those from the negative binomial model, supporting the robustness of our main findings. However, the hurdle model treats zero episodes as arising from a distinct process, an assumption that does not reflect the epidemiological nature of URTI. We therefore retained the negative binomial model as the primary analytic approach (Supplementary materials). Because all participants had the same 12-month recall period, the adjusted IRR was estimated as the ratio of annualized episode counts between groups.

Adjusted IRRs and 95% confidence intervals (CIs) from Model 3 were displayed in forest plots generated with the ggplot2 and patchwork packages in R. To test whether the entry order affected the comparison between variable groups, we repeated the hierarchical analysis after reversing the entry order of lifestyle factors and protective behaviors.

To assess whether daytime sleepiness modified the association between natural ventilation and URTI episodes, the “almost never” and “occasionally” ventilation categories were combined into a low-frequency ventilation reference group because some cross-classified subgroups contained no observations. Interaction terms between daytime sleepiness and natural ventilation were then added to Model 3. An LRT compared models with and without the interaction terms. Regression results are reported as IRRs with 95% CIs. All tests were two-sided, and *p* < 0.05 was considered statistically significant.

## 3 Results

### 3.1 Participant characteristics

The analytic sample included 1,535 Chinese college students with a history of SARS-CoV-2 infection. The sample comprised 754 males (49.12%) and 781 females (50.88%). Most participants were 17–18 years old (1,151, 74.98%), and 384 (25.02%) were 19–30 years old. According to the Chinese BMI classification, 283 participants (18.44%) were underweight, 842 (54.85%) had normal weight, 298 (19.41%) were overweight, and 112 (7.30%) had obesity. Most students (1,519, 98.96%) reported no chronic diseases. Most students have received at least 2 doses of the COVID-19 vaccine (1,431, 93.22%) (Table 1).

**Table 1:** Characteristics of the analytic sample and univariate associations with URTI episodes (*N* = 1,535).

Variable	Level	*n* (%)	URTI episodes (times/year) Median (P25, P75)	Test statistic	*p-*value
Demographics					
Sex				−6.671	<0.001^***^
Male		754(49.12)	1 (1, 3)		
Female		781(50.88)	2 (1, 4)		
Age (years)				−1.813	0.070
17–18		1,151 (74.98)	1 (1, 4)		
19–30		384 (25.02)	1 (1, 4)		
BMI(kg/m^2^)				−0.032	0.975
<18.5		283 (18.44)	1 (1, 4)		
18.5–23.9		842 (54.85)	1 (1, 4)		
24–27.9		298 (19.41)	1 (1, 4)		
≥28		112 (7.30)	1 (1, 4)		
Chronic diseases				−3.907	<0.001^***^
No		1,519 (98.96)	1 (1, 4)		
Yes		16 (1.04)	4 (3.75, 4)		
COVID-19 vaccination doses				−2.079	0.038^*^
≤1		104 (6.78)	0 (0,2)		
≥2		1,431 (93.22)	0 (0,3)		
Lifestyle					
Smoking				−2.677	0.007^**^
No		1,481 (96.48)	1 (1, 4)		
Yes		54 (3.52)	3 (1, 5)		
Alcohol use				−0.463	0.643
No		1,503 (97.92)	1 (1, 4)		
Yes		32 (2.08)	1.5 (1, 4.5)		
Daytime sleepiness(day/week)				10.560	<0.001^***^
0		350 (22.80)	1 (1, 1)		
<1		357 (23.26)	1 (1, 4)		
1–2		350 (22.80)	2 (1, 4)		
≥3		478 (31.14)	3 (1, 4)		
Sleep duration (hours)				−6.560	<0.001^***^
<6		141 (9.19)	3 (1, 4)		
6- < 7		716 (46.64)	2 (1, 4)		
7- < 8		541 (35.24)	1 (1, 3)		
≥8		137 (8.93)	1 (1, 3)		
Sedentary behavior				−6.866	<0.001^***^
No		621 (40.46)	1 (1, 3)		
Yes		914 (59.54)	2 (1, 4)		
Light-intensity physical activity (min/day)				−1.805	0.071
0–9		232 (15.11)	2 (1, 4)		
10–19		339 (22.08)	1 (1, 4)		
20–29		410 (26.71)	1 (1, 4)		
30–39		196 (12.77)	1 (1, 4)		
≥40		358 (23.32)	1 (1, 4)		
Moderate-intensity physical activity (min/day)				−4.365	<0.001^***^
0–9		429 (27.95)	2 (1, 4)		
10–19		454 (29.58)	1 (1, 4)		
20–29		339 (22.08)	1 (1, 4)		
≥30		313 (20.39)	1 (1, 3)		
Vigorous-intensity physical activity (min/day)				−2.175	0.030^*^
0–9		551 (35.90)	2 (1, 4)		
10–19		475 (30.94)	1 (1, 4)		
20–29		302 (19.67)	1 (1, 4)		
≥30		207 (13.49)	1 (1, 3)		
Protective behaviors					
Handwashing before meals				−2.649	0.008^**^
Almost never		78 (5.08)	1 (1, 4)		
Occasionally		280 (18.24)	2 (1, 4)		
Sometimes		149 (9.71)	2 (1, 4)		
Often		349 (22.74)	1 (1, 4)		
Always		679 (44.23)	1 (1, 4)		
Handwashing after restroom use				1.333	0.183
Almost never or occasionally		25(1.63)	1 (1, 3)		
Sometimes		31 (2.02)	3 (1, 4)		
Often		150 (9.77)	1 (1, 3)		
Always		1,329 (86.58)	1 (1, 4)		
Mask wearing indoors				−3.465	0.001^**^
Almost never		492 (32.05)	2 (1, 4)		
Occasionally		582 (37.91)	1 (1, 4)		
Sometimes		148 (9.64)	3 (1, 4)		
Often		127 (8.27)	1 (1, 2.5)		
Always		186 (12.12)	1 (1, 3)		
Maintaining 1-meter distance				−4.580	<0.001^***^
Almost never		302 (19.67)	2 (1, 4)		
Occasionally		492 (32.05)	1 (1, 4)		
Sometimes		213 (13.88)	2 (1, 4)		
Often		300 (19.54)	1 (1, 4)		
Always		228 (14.85)	1 (1, 3)		
Avoiding touching the eyes, mouth, or nose				−4.235	<0.001^***^
Almost never		217 (14.14)	2 (1, 4)		
Occasionally		459 (29.90)	2 (1, 4)		
Sometimes		315 (20.52)	2 (1, 4)		
Often		267 (17.39)	1 (1, 3.5)		
Always		277 (18.05)	1 (1, 3)		
Avoiding contact with public surfaces				−3.589	<0.001^***^
Almost never		180 (11.73)	1 (1, 4)		
Occasionally		407 (26.51)	2 (1, 4)		
Sometimes		266 (17.33)	2 (1, 4)		
Often		349 (22.74)	1 (1, 4)		
Always		333 (21.69)	1 (1, 3)		
Disinfecting phones and keys (times/week)				−4.303	<0.001^***^
0		273 (17.79)	2 (1, 4)		
1–2		458 (29.84)	2 (1, 4)		
3–4		220 (14.33)	2 (1, 4)		
5–6		304 (19.80)	1 (1, 4)		
≥7		280 (18.24)	1 (1, 3)		
Opening windows for natural ventilation(30 min/session)				−5.670	<0.001^***^
Almost never		39 (2.54)	4 (1, 6)		
Occasionally		246 (16.03)	1 (1, 4)		
Once daily		262 (17.07)	3 (1, 4)		
1–2 times daily		452 (29.45)	1 (1, 4)		
≥Twice daily		536 (34.92)	1 (1, 3)		

Data are presented as *n* (%) for categorical variables and as median (25th percentile, 75th percentile) for URTI episodes. *p*-values were calculated using the Mann–Whitney *U* test for binary variables and the Jonckheere–Terpstra test for ordered categorical variables.

*p < 0.05; **p < 0.01; ***p < 0.001.

### 3.2 URTI episodes during the 12 months before the survey

The median number of URTI episodes was 0 (interquartile range, 0–3). More than half of the students (53.0%) reported no episodes during the previous year. The highest reported frequency among students with at least one episode was 12 episodes/year (Figure 1).

**Figure 1:**
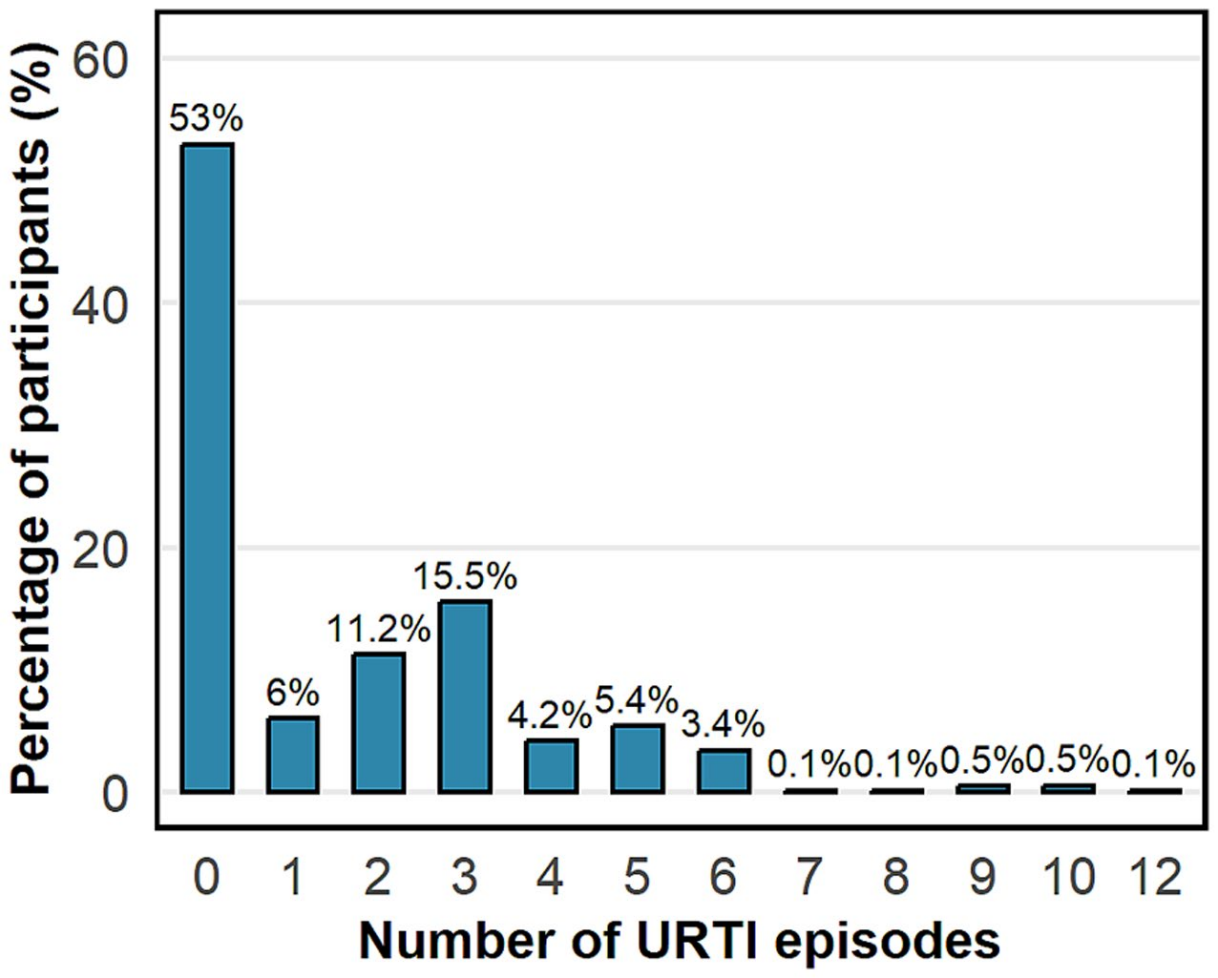
Distribution of URTI episodes during the 12 months before the survey.

### 3.3 Univariate analysis of factors associated with URTI episodes

Univariate analyses were used to describe the association between each variable and the number of URTI episodes. All variables were subsequently included in the multivariable models, regardless of their univariate significance.

*Demographic factors*: The Mann–Whitney *U* test showed differences in the number of URTI episodes by sex, chronic diseases and COVID-19 vaccination doses (all *p* < 0.05; Table 1), but not by age (*p* > 0.05). The Jonckheere–Terpstra test showed no significant trend across BMI categories (*p* > 0.05).

*Lifestyle factors*: The Mann–Whitney *U* test showed differences in URTI episodes by smoking status and sedentary behavior (both *p* < 0.05; Table 1), but not by alcohol use (*p* > 0.05).

For ordered lifestyle variables, the Jonckheere–Terpstra test showed a positive trend between daytime sleepiness and URTI episodes (*p* for trend < 0.001; Table 1) and inverse trends for sleep duration and moderate- and vigorous-intensity physical activity (all *p* for trend < 0.05). No significant trend was observed for light-intensity physical activity (*p* > 0.05).

*Protective behaviors*: The Jonckheere–Terpstra test showed no significant trend for handwashing after restroom use (*p* > 0.05). Inverse trends were observed for all other protective behaviors, including handwashing before meals, mask wearing indoors, maintaining 1-meter distance, avoiding touching the eyes, mouth, or nose and public surfaces, disinfecting phones and keys, and opening windows for natural ventilation (all *p* for trend < 0.05; Table 1).

### 3.4 Hierarchical negative binomial regression models: lifestyle factors versus protective behaviors

Hierarchical negative binomial regression was used to assess the independent associations of lifestyle factors and protective behaviors with URTI episodes. As shown in Table 2, Model 1, which included demographic variables, had a Nagelkerke pseudo-*R*^2^ of 0.031. Adding lifestyle factors in Model 2 increased the pseudo-*R*^2^ to 0.118. Adding protective behaviors in Model 3 further increased it to 0.149, indicating that both variable groups contributed additional information.

**Table 2:** Hierarchical negative binomial regression of factors associated with URTI episodes.

Variables	Model 1 (Demographics)	Model 2 (+ Lifestyle)	Model 3 (+Protective behaviors)
	IRR (95% CI)	IRR (95% CI)	IRR (95% CI)
Demographics			
Sex			
Female	1.672 (1.450–1.927)^***^	1.467 (1.273–1.690)^***^	1.386 (1.186–1.621)^***^
Age(years)			
19–30	0.920 (0.783–1.081)	0.900 (0.764–1.061)	0.868 (0.727–1.036)
BMI (kg/m^2^)			
18.5–23.9	1.107 (0.926–1.323)	1.211 (1.016–1.444)^*^	1.226 (1.017–1.478)^*^
24.0–27.9	1.341 (1.057–1.703)^*^	1.408 (1.120–1.769)^**^	1.399 (1.099–1.781)^**^
≥28.0	1.253 (0.923–1.700)	1.413 (1.044–1.911)^*^	1.532 (1.106–2.122)^*^
Chronic diseases			
Yes	1.896 (1.476–2.434)^***^	1.347 (1.078–1.684)^**^	1.583 (1.153–2.173)^**^
COVID-19 vaccination doses			
≥2	1.455 (1.064–1.990)^*^	1.490 (1.090–2.038)^*^	1.523 (1.095–2.118)^*^
Lifestyle			
Smoking			
Yes	—	1.243 (0.830–1.861)	1.252 (0.775–2.020)
Drinking			
Yes	—	1.135 (0.587–2.193)	1.233 (0.562–2.705)
Daytime sleepiness(day/week)			
<1	—	1.950 (1.500–2.536)^***^	1.768 (1.325–2.359)^***^
1–2	—	2.592 (2.011–3.340)^***^	2.324 (1.776–3.041)^***^
≥3	—	2.525 (1.957–3.259)^***^	2.319 (1.772–3.034)^***^
Sleep duration			
6–<7 h	—	0.943 (0.749–1.189)	0.880 (0.694–1.116)
7–<8 h	—	0.662 (0.515–0.851)^**^	0.613 (0.465–0.808)^***^
≥8 h	—	0.676 (0.466–0.981)^*^	0.644 (0.449–0.925)^*^
Sedentary behavior			
Yes	—	1.255 (1.078–1.460)^**^	1.220 (1.029–1.445)^*^
Light PA			
10–19 min/day	—	0.924 (0.730–1.169)	1.050 (0.821–1.343)
20–29 min/day	—	1.248 (0.964–1.616)	1.483 (1.138–1.932)^**^
30–39 min/day	—	0.940 (0.712–1.240)	1.163 (0.871–1.553)
≥40 min/day	—	1.093 (0.843–1.417)	1.323 (1.001–1.748)^*^
Moderate PA			
10–19 min/day	—	0.651 (0.529–0.800)^***^	0.633 (0.511–0.785)^***^
20–29 min/day	—	0.706 (0.548–0.910)^**^	0.637 (0.488–0.831)^***^
30–39 min/day	—	0.576 (0.440–0.754)^***^	0.523 (0.396–0.690)^***^
Vigorous PA			
10–19 min/day	—	1.312 (1.085–1.588)^**^	1.286 (1.062–1.558)^*^
20–29 min/day	—	1.317 (1.033–1.680)^*^	1.330 (1.034–1.710)^*^
30–39 min/day	—	1.597 (1.202–2.121)^**^	1.611 (1.198–2.167)^**^
Protective behaviors			
Handwashing before meals			
Occasionally	—	—	0.908 (0.634–1.302)
Sometimes	—	—	1.184 (0.791–1.771)
Often	—	—	0.964 (0.666–1.397)
Always	—	—	0.982 (0.674–1.430)
Handwashing after restroom use			
Sometimes	—	—	1.679 (0.781–3.608)
Often	—	—	2.602 (1.334–5.075)^**^
Always	—	—	2.407 (1.282–4.519)^**^
Mask wearing indoors			
Occasionally	—	—	1.006 (0.829–1.221)
Sometimes	—	—	1.408 (1.077–1.840)^*^
Often	—	—	0.666 (0.453–0.978)^*^
Always	—	—	0.932 (0.627–1.386)
Maintaining 1-meter distance			
Occasionally	—	—	1.105 (0.874–1.397)
Sometimes	—	—	1.119 (0.851–1.471)
Often	—	—	1.132 (0.873–1.467)
Always	—	—	1.011 (0.669–1.528)
Avoiding touching the eyes, mouth, or nose			
Occasionally	—	—	0.846 (0.634–1.129)
Sometimes	—	—	0.816 (0.580–1.147)
Often	—	—	1.048 (0.737–1.492)
Always	—	—	0.944 (0.656–1.359)
Avoiding contact with public surfaces			
Occasionally	—	—	1.390 (0.962–2.009)
Sometimes	—	—	1.354 (0.905–2.026)
Often	—	—	1.344 (0.900–2.009)
Always	—	—	1.463 (0.961–2.227)
Disinfecting phones and keys			
1–2 times/week	—	—	1.007 (0.765–1.325)
3–4 times/week	—	—	1.162 (0.875–1.542)
5–6 times/week	—	—	0.937 (0.700–1.255)
7 times/week	—	—	0.722 (0.511–1.018)
Opening windows for natural ventilation			
Occasionally	—	—	0.475 (0.311–0.725)^***^
Once daily	—	—	0.466 (0.311–0.696)^***^
1–2 times daily	—	—	0.425 (0.279–0.648)^***^
≥Twice daily	—	—	0.431 (0.285–0.651)^***^

Model fit	Demographics	+ Lifestyle	+ Protective behaviors
AIC	5082.8	4981.4	4991.5
Nagelkerke *R*^2^	0.031	0.118	0.149
Likelihood ratio test (vs. previous model)	—	*χ*^2^(19) = 139.43^***^	χ^2^(31) = 51.88^*^

Model 1 was adjusted for demographic factors.

Model 2 was additionally adjusted for lifestyle factors.

Model 3 was additionally adjusted for protective behaviors.

All variance inflation factor values (VIF) were <3, indicating no evidence of important multicollinearity.

*p*-values and 95% CIs for variables were derived from heteroskedasticity-robust standard errors (HC3).

IRR, incidence rate ratio; CI, confidence interval; AIC, Akaike information criterion; BMI, body mass index; PA, physical activity; VIF, variance inflation factor.

Reference categories were male sex; age 17–18 years; BMI <18.5 kg/m^2^; no chronic diseases; COVID-19 vaccination doses ≤1; no smoking; no alcohol use; no daytime sleepiness; sleep duration <6 h; no sedentary behavior; 0–9 min/day for each PA intensity; and “almost never” for each protective behavior, except disinfecting phones and keys (0 times/week) and handwashing after restroom use (“almost never” and “occasionally” combined).

*p < 0.05; **p < 0.01; ***p < 0.001.

The likelihood ratio test (LRT) showed that the addition of lifestyle factors significantly improved model fit (*χ*^2^ = 139.43, df = 19, *p* < 0.001), and the further inclusion of protective behaviors also yielded a significant improvement (*χ*^2^ = 51.88, df = 31, *p* = 0.011). However, the effect size per degree of freedom was notably smaller (*χ*^2^/df = 1.67) compared to that of lifestyle factors (7.34), and the AIC was less favorable (4991.5 vs. 4981.4). These findings suggest that, in our model, lifestyle factors were more strongly associated with URTI episodes than protective behaviors.

### 3.5 Multivariable analysis of factors associated with URTI episodes

In Model 3, several demographic, lifestyle, and protective-behavior variables were independently associated with the number of URTI episodes.

*Demographic factors*: Female sex, higher BMI categories, chronic diseases and COVID-19 vaccination ≥2 doses were associated with more URTI episodes (all *p* < 0.05; Figure 2A). Age was not significantly associated with URTI episodes (*p* > 0.05).

**Figure 2:**
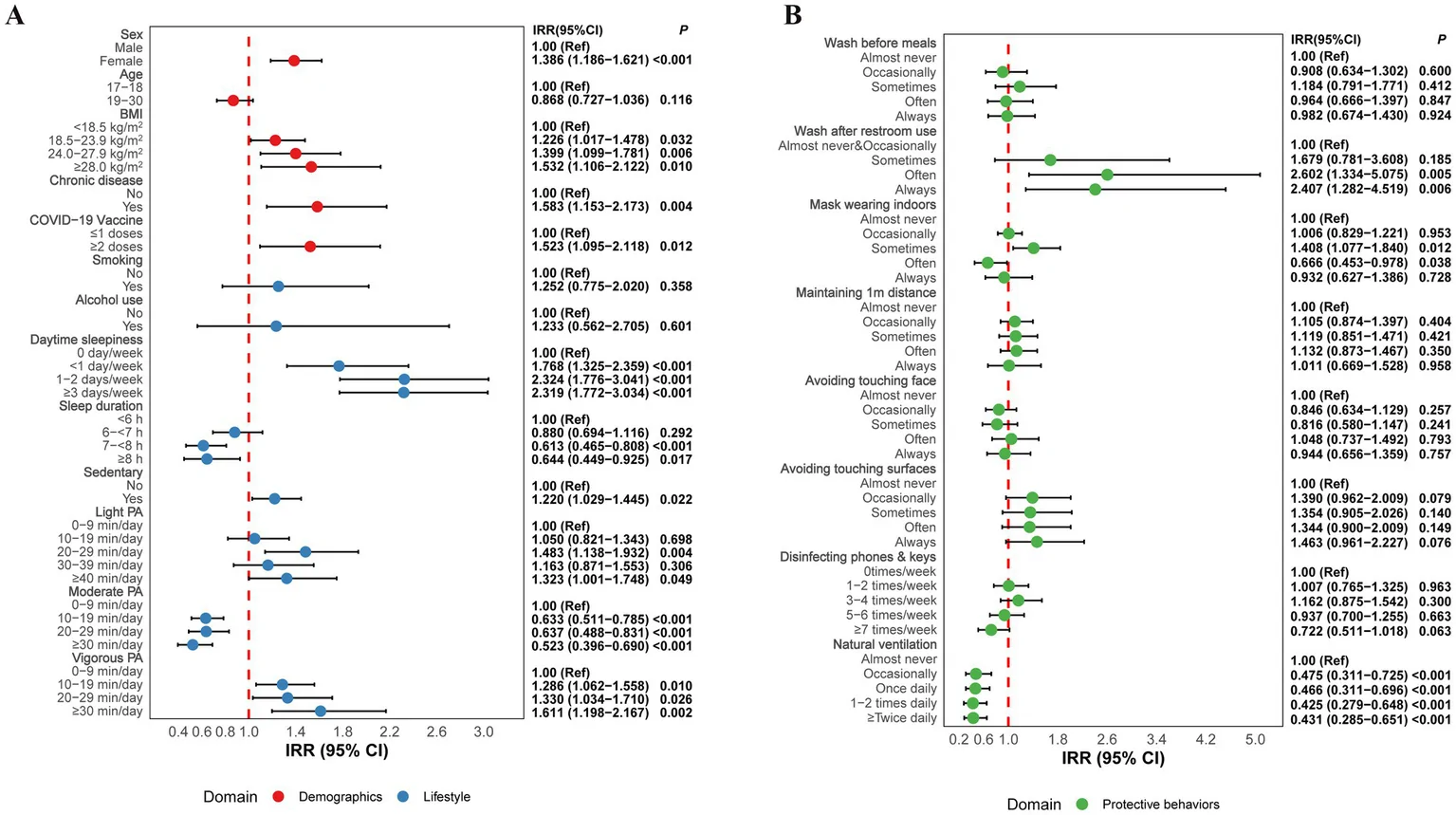
**(A)** Forest plot of adjusted IRRs and 95% CIs for demographic and lifestyle factors associated with URTI episodes during the 12 months before the survey (Model 3). An IRR > 1 indicates more URTI episodes, whereas an IRR < 1 indicates fewer episodes. Error bars represent 95% CIs. PA, physical activity. **(B)** Forest plot of adjusted IRRs and 95% CIs for protective behaviors associated with URTI episodes during the 12 months before the survey (Model 3). An IRR > 1 indicates more URTI episodes, whereas an IRR < 1 indicates fewer episodes. Error bars represent 95% CIs. Wash before meals, handwashing before meals; wash after restroom, handwashing after restroom use; maintaining 1 m distance, maintaining 1-meter distance; avoiding touching face, avoiding touching the eyes, mouth, or nose; avoiding touching surfaces, avoiding contact with public surfaces; disinfecting phones & keys: disinfecting phones and keys; natural ventilation, opening windows for natural ventilation.

*Lifestyle factors*: Daytime sleepiness, sedentary behavior, vigorous-intensity physical activity, and light-intensity physical activity (20–29 and ≥40 min/day) were associated with more URTI episodes (all *p* < 0.05; Figure 2A). Sleep duration of at least 7 h and moderate-intensity physical activity were associated with fewer episodes (all *p* < 0.05). Smoking and alcohol use were not significantly associated with URTI episodes (both *p* > 0.05).

*Protective behaviors*: All natural ventilation categories and mask wearing indoors “often” were associated with fewer URTI episodes (all *p* < 0.05; Figure 2B). Mask wearing indoors “sometimes” and handwashing after restroom use “often” or “always” were associated with more episodes (all *p* < 0.05). Handwashing before meals, maintaining 1-meter distance, avoiding touching the eyes, mouth, or nose and public surfaces, and disinfecting phones and keys were not significantly associated with URTI episodes (all *p* > 0.05).

Detailed IRRs and 95% CIs are presented in Table 2. Figure 2A shows the estimates for demographic and lifestyle factors, Figure 2B shows the estimates for protective behaviors, and Figure 3 summarizes the independently associated factors.

**Figure 3:**
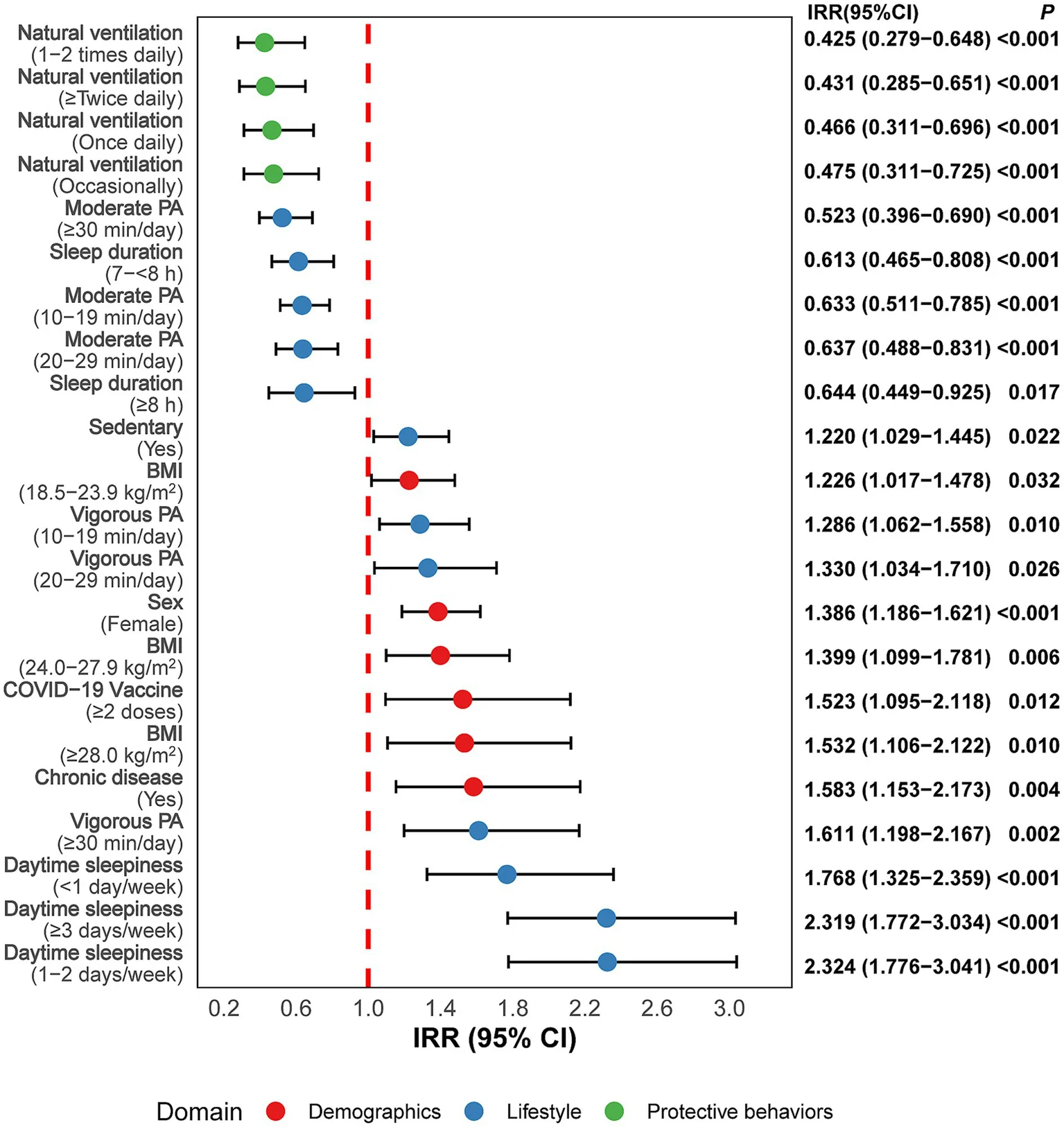
Forest plot of adjusted IRRs and 95% CIs for factors independently associated with URTI episodes during the 12 months before the survey (Model 3). An IRR > 1 indicates more URTI episodes, whereas an IRR < 1 indicates fewer episodes. Error bars represent 95% CIs. Natural ventilation, opening windows for natural ventilation; PA, physical activity.

### 3.6 Sensitivity analysis

In the sensitivity analysis, protective behaviors were entered before lifestyle factors. When protective behaviors were added to the demographic model (Model 4), the Nagelkerke pseudo-*R*^2^ was 0.080, the incremental *χ*^2^/df was 2.48 (*p* < 0.001 vs. Model 1), and the AIC was 5067.9. After lifestyle factors were added, the pseudo-*R*^2^ increased to 0.149, the incremental *χ*^2^/df was 6.02 (*p* < 0.001), and the AIC decreased to 4991.5 (ΔAIC = −76.4 vs. Model 4). These results were consistent with the primary analysis and supported the stronger overall association of lifestyle factors with URTI episodes.

### 3.7 Interaction analysis

The model containing interaction terms between daytime sleepiness and natural ventilation did not fit significantly better than the main-effects model (LRT: *χ*^2^ = 13.152, df = 8, *P* for interaction = 0.107). Thus, there was no statistical evidence that daytime sleepiness modified the association between natural ventilation and URTI episodes.

## 4 Discussion

Three main findings emerged from this study. First, we quantified the relative contributions of lifestyle factors and protective behaviors to URTI episodes within a hierarchical analytical framework, an approach that has rarely been used in previous studies. We found that both lifestyle factors and protective behaviors were independently associated with URTI episodes. Second, lifestyle factors consistently showed stronger associations with URTI episodes than protective behaviors, and these associations persisted when the entry order was reversed. This finding does not negate the value of protective behaviors. Instead, it suggests a hierarchical difference in their roles. Lifestyle factors may be related to baseline host defenses against pathogens (9, 10), whereas protective behaviors mainly reduce exposure to pathogens. Third, natural ventilation was the only protective behavior that showed a consistent inverse association, implying that environmental interventions may offer advantages over individual-level behavioral measures in real-world campus environments. The following section discusses the main findings for sex, BMI, COVID-19 vaccination, sleep, physical activity, sedentary behavior, and natural ventilation, together with their public health implications.

Several demographic and lifestyle factors were associated with URTI episodes. Females had a 39.2% higher adjusted rate of URTI episodes than males, consistent with previous reports. Possible explanations include sex-related differences in immune regulation and higher psychosocial stress (25, 26). Some studies have reported a U-shaped association, in which both low and high BMI are associated with increased infection risk (27, 28). In contrast, our data were consistent only with the ascending portion of this pattern. Overweight and obesity are associated with chronic systemic inflammation, altered immune-cell metabolism (29), and delayed protective mucosal immune responses in the airways (30). The apparently lower URTI episode rate in underweight participants relative to normal-weight participants should be interpreted cautiously. This finding may partly reflect unmeasured behavioral differences, such as social contact patterns and pathogen exposure (31), as well as differences in study populations.

Regarding vaccination, contrary to our expectation that COVID-19 vaccination would be associated with fewer URTI episodes through protecting against SARS-CoV-2, we found that ≥2 doses were associated with more URTI episodes. This finding is consistent with a Korean nationwide study (*N* = 51.6 million), which reported that ≥4 doses were associated with a higher risk of URI (aHR = 1.32, 95% CI: 1.32–1.33) (32). One possible explanation is non-specific immune effects of vaccines, as has also been reported after influenza vaccination. For example, children who received inactivated influenza vaccine had an increased risk of non-influenza respiratory infections (33, 34). Of note, this observation does not negate the well-established protective effects of COVID-19 vaccines against severe disease and mortality. However, the cross-sectional design of the present study does not permit causal or mechanistic inference. Further research is warranted to clarify the immunological basis of this observation.

Compared with sleep duration of <6 h, 6–<7 h was not significantly associated with URTI episodes, whereas sleep duration of at least 7 h was associated with fewer episodes (IRR, 0.613–0.644), consistent with previous research (35). Studies have also linked very long sleep duration to underlying illness and higher infection risk, but this pattern was not observed in our generally healthy college-student sample. Daytime sleepiness showed a stronger association than reported sleep duration. Compared with no daytime sleepiness, sleepiness at least once per week was associated with a 76.8–132.4% higher adjusted rate of URTI episodes (IRR, 1.768–2.324), representing the largest effect size among all lifestyle factors examined, in line with earlier findings (9, 36). Daytime sleepiness may be a more sensitive marker of poor sleep quality than sleep duration alone. A bidirectional relation between daytime sleepiness and inflammation may contribute to this association (37, 38), because chronic inflammation can impair respiratory mucosal barriers and immune defense (39). Overall, at least 7 h of sleep and less frequent daytime sleepiness were associated with fewer URTI episodes.

Sedentary behavior and vigorous-intensity physical activity were associated with more URTI episodes, whereas moderate-intensity physical activity was associated with fewer episodes. Light-intensity activity showed no consistent dose–response pattern. These findings are broadly consistent with the J-shaped model proposed in exercise immunology (40, 41). However, the elevated rate in the low-intensity groups (20–29 and ≥40 min/day) may reflect reverse causality, as students in poor health may choose low-intensity activities. Moderate- and vigorous-intensity activities require better health and are less susceptible to this bias. Associations were strongest in the highest categories of moderate- and vigorous-intensity activity (IRR, 0.523 and 1.611, respectively), although the cross-sectional design does not support interpreting these categories as causal thresholds. Sedentary behavior may reflect insufficient regular activity and is associated with impaired metabolic and inflammatory profiles (42). Moderate-intensity activity may support immune surveillance and anti-inflammatory responses (40, 43). By contrast, repeated strenuous activity combined with inadequate recovery may transiently alter immune-cell distribution and function (10, 44–46). Campus health programs should therefore support regular moderate-intensity activity, reduce prolonged sedentary time, and advise students to balance vigorous exercise with sufficient recovery.

Natural ventilation was the only protective behavior with a consistent inverse association across frequency categories and had the lowest IRRs among all factors (0.425–0.475). The interaction analysis found no evidence that this association differed by the level of daytime sleepiness (*p* = 0. 107). Ventilation can dilute the concentration of airborne pathogens (47), and lower indoor carbon dioxide concentrations may reduce conditions that favor viral aerostability (48). A model based on the Wells–Riley equation suggested that classroom cross-ventilation through open windows may provide greater protection than single-sided ventilation combined with mask use (49). Other evidence also supports the value of environmental controls for reducing airborne exposure (50). In contrast, mask wearing and handwashing after restroom use showed inconsistent associations across categories. These results should not be interpreted as evidence that these behaviors increase infection risk. Reverse causality is possible because students with recurrent respiratory symptoms may adopt personal protective behaviors more frequently. Natural ventilation is often implemented at the classroom or institutional level and may be less responsive to individual risk perception. Because respiratory pathogens are transmitted mainly through droplets and aerosols rather than contact alone (51), ventilation may also be more directly related to URTI occurrence than contact-focused measures. Natural ventilation is low cost and easy to implement, supporting its use as a campus-level measure.

Demographic characteristics and long-term lifestyle patterns may be less responsive to recent perceptions of infection risk than personal protective behaviors. Associations for protective behaviors may therefore be more affected by reverse causality in a cross-sectional study. Taken together, the findings support a two-part campus strategy: long-term health measures, including not being overweight or obese, good-quality sleep, and appropriate physical activity, combined with exposure-reduction measures, particularly natural ventilation.

### 4.1 Limitations

This study has several limitations. First, the cross-sectional design does not establish temporal or causal relations, and reverse causality may explain some associations. Second, although the outcome data included both self-reported and physician-diagnosed cases, all exposure measures and the self-reported component of the outcome may be subject to recall and reporting bias. We used quarterly recall to reduce error in the reported number of URTI episodes, but the episodes were not confirmed by clinical examination or pathogen testing. Third, residual confounding is possible because influenza vaccination status, dietary, social contact factors and other potential determinants were not measured. Fourth, participants were recruited from universities in Jiangsu Province. Although the sample included several types of universities and students from different parts of China, the findings may not apply directly to all college students or to populations outside China.

## 5 Conclusion

In this cross-sectional study, lifestyle factors showed stronger overall associations with URTI episodes than protective behaviors. Female sex, overweight or obesity, daytime sleepiness, sedentary behavior, and vigorous-intensity physical activity were associated with more URTI episodes. Moderate-intensity physical activity, sleep duration of at least 7 h, and natural ventilation were associated with fewer episodes. Natural ventilation was the only protective behavior with a consistent inverse association. These findings support campus health programs centered on long-term lifestyle measures, with environmental controls serving as supportive measures.

## Data Availability

The raw data supporting the conclusions of this article will be made available by the authors, without undue reservation.
