# Supplementary material for: Lifestyle factors or protective behaviors: which are more strongly associated with upper respiratory tract infections after COVID-19? A cross-sectional study of 1,535 Chinese college students
Source: Front Public Health. 2026 Sep 23;14:1958833. doi: 10.3389/fpubh.2026.1958833 (PMC13647571; doi:10.3389/fpubh.2026.1958833)
Supplement: Supplementary file 1 [file Supplementary_file_1.DOCX]

**Model Selection and Fit**

The outcome was a count variable with a variance-to-mean ratio of 2.68 and a substantial proportion of zeros (53.0%). We selected a negative binomial regression model as the primary analytic approach to accommodate potential overdispersion. Model fit was evaluated using simulation-based residual diagnostics from the DHARMa package. The Q-Q plot (Figure S1A) showed that most residuals closely followed the diagonal line, although the Kolmogorov–Smirnov test was statistically significant (*P* = 0.00863). This deviation was small and unlikely to affect inference given the large sample size (n = 1,535). No influential outliers were detected (outlier frequency = 0.065%, *P* =0.470).

 The residuals-versus-predicted plot (Figure S1B) showed slight deviations in the extreme tails, as indicated by the red curves. The dispersion test indicated mild underdispersion (dispersion = 0.591, *P* < 0.001), suggesting some variance compression, possibly due to the high proportion of zeros in the data. To ensure robust inference, we applied HC3 heteroskedasticity-robust standard errors to Models 1–3. All reported 95% CIs and P values were based on these robust standard errors.

The zero-inflation test for the negative binomial model was significant (ratioObsSim = 1.119, p < 0.001), indicating that the observed zeros were 11.9% higher than expected. However, such deviations are common in count data studies and are unlikely to substantially affect our main findings. To further examine the impact of zero inflation, we also fitted a hurdle model as a sensitivity analysis. The hurdle model treats zero and positive counts as arising from two separate processes: a binomial logistic component models the probability of any URTI episode (zero vs. positive), and a truncated negative binomial component models the episode count among those with at least one episode. The hurdle model showed a lower AIC than the negative binomial model (4756 vs. 4991.5), indicating better fit. However, the key findings including the protective effect of natural ventilation and the risk associations for female sex, daytime sleepiness, and vigorous physical activity remained consistent across both models. We retained the negative binomial model as the primary analytic approach, based on its greater alignment with the biological nature of URTI episode counts and its more direct interpretability.

A	B

Figure S1 Diagnostic plots for the negative binomial regression model (Model 3).  (A) QQ plot of simulated residuals. (B) Residuals vs. fitted values.
